# Comparison of techniques for corneal epithelium cell culture for the collection of conditioned medium

**DOI:** 10.5935/0004-2749.2022-0084

**Published:** 2024-04-03

**Authors:** Magda Massae Hata Viveiros, Luís Henrique Zucoloto, Álvio Issao Shiguematsu, Cláudia Aparecida Rainho, Silvana Artioli Schellini

**Affiliations:** 1 Department of Ophthalmology, Faculdade de Medicina de Botucatu, Universidade Estadual Paulista “Júlio de Mesquita Filho,” Botucatu, SP, Brazil; 2 Faculdade de Medicina de Botucatu, Universidade Estadual Paulista “Júlio de Mesquita Filho,” Botucatu, SP, Brazil; 3 Department of Chemical and Biological Sciences, Instituto de Biociências de Botucatu, Universidade Estadual Paulista “Júlio de Mesquita Filho” Botucatu, SP, Brazil

**Keywords:** Cell culture, Mesenchymal stem cells, Cell differentiation, Epithelial cells, Cornea, Conditioned culture medium, Culture techniques, Cultivo de células, Células tronco mesenquimais, Diferenciação celular, Células epiteliais, Córnea, Meio de cultivo condicionado, Técnicas de cultivo

## Abstract

**Purposes:**

To determine the best protocol in obtaining the higher yield of conditioned culture medium to be used for the bone marrow mesenchymal stem cell differentiation into corneal epithelial cells, five techniques for the primary culture of human corneal epithelial cells were evaluated.

**Methods:**

The studied culture techniques of corneal epithelial cells were: explants in culture flasks with and without hydrophilic surface treatment, on amniotic membrane, with enzymatic digestion, and by corneal scraping. The conditioned culture medium collected from these cultures was used to differentiate human bone marrow mesenchymal stem cells into corneal epithelial cells, which were characterized using flow cytometry with pan-cytokeratin and the corneal-specific markers, cytokeratin 3 and cytokeratin 12.

**Results:**

The culture technique using flasks with hydrophilic surface treatment resulted in the highest yield of conditioned culture medium. Flasks without surface treatment resulted to a very low success rate. Enzymatic digestion and corneal scraping showed contamination with corneal fibroblasts. The culture on amniotic membranes only allowed the collection of culture medium during the 1st cell confluence. The effectiveness of cell differentiation was confirmed by cytometry analysis using the collected conditioned culture medium, as demonstrated by the expressions of cytokeratin 3 (95.3%), cytokeratin 12 (93.4%), and pan-cytokeratin (95.3%).

**Conclusion:**

The culture of corneal epithelial cell explants in flasks with hydrophilic surface treatment is the best technique for collecting a higher yield of conditioned culture medium to be used to differentiate mesenchymal stem cells.

## Abstract (pt)

**Objetivos:**

Foram estudadas cinco técnicas de cultivo primário de células epiteliais de córnea humana para se determinar o melhor protocolo para a obtenção do maior rendimento de meio de cultivo condicionado para ser utilizado na diferenciação de células tronco mesenquimais para células epiteliais de córnea.

**Métodos:**

As técnicas de cultivo estudadas foram: explantes em frascos de cultivo com e sem tratamento hidrofílico de superfície, sobre membrana amniótica, com digestão enzimática e por raspado de córnea. O meio de cultivo condicionado foi coletado e as células tronco mesenquimais induzidas a se diferenciarem em células epiteliais da córnea utilizando o meio de cultivo condicionado. As células foram caracterizadas por citometria de fluxo com pan-citoqueratina e com os marcadores específicos da córnea, citoqueratina 3 e citoqueratina 12.

**Resultados:**

A técnica utilizando frascos com o tratamento de superfície apresentou o maior rendimento de meio de cultivo condicionado. Os frascos sem tratamento de superfície levaram a uma taxa de sucesso muito baixa. A digestão enzimática e a raspagem da córnea mostraram contaminação das culturas com fibroblastos de córnea. A cultura sobre membranas amnióticas só permitiu a coleta do meio de cultivo condicionado durante a 1ª confluência celular. A análise de citometria de fluxo confirmou o sucesso da diferenciação celular utilizando o meio de cultivo condicionado coletado, demonstrada pela expressão de citoqueratina 3 (95,3%), citoqueratina 12 (93,4%) e pan-citoqueratina (95,3%).

**Conclusão:**

O cultivo de explantes de células tronco mesenquimais em frascos com tratamento hidrofílico de superfície é a melhor técnica para a obtenção de um alto rendimento de meio de cultivo condicionado.

## INTRODUCTION

To recover transparency of injured cornea^(1)^, advances in regenerative medicine are focused on corneal cell transference. Mesenchymal stem cells (MSCs) are currently the subject of great scientific interest, particularly in the field of ocular surface regeneration. MSCs are a type of multipotent progenitor cells^(2)^, which have the ability to auto-regenerate as undifferentiated cells, and with the possibility to differentiate into lineages of mesenchymal tissues such as bone, cartilage, fat, muscle^(3,4)^, cardiac muscle^(5)^, neurons^(6)^, and corneal epithelial cells (CEC)^(7-11)^. Autologous MSCs can be collected, expanded, and differentiated using in vitro techniques and were successfully applied.

Cellular differentiation can be induced by conditioned culture medium (CM). When conditioning cells are cultivated, they secrete mediator substances into the cultivated medium, which can be preserved after the original conditioning cells are removed, generating the CM. This CM contains conditioning cell mediator substances, and when used on MSCs, these cells can develop the characteristics of the conditioning cells^(12)^. To induce differentiation, it is essential to use a cell culture technique that allows rapid cell proliferation and a high yield of CM from the conditioning cell cultures.

This study aimed to evaluate five techniques of primary CEC culture, to determine the protocol with the highest yield of CM from these cultures to be used in MSC differentiation into CEC.

## METHODS

### Corneal epithelial cell primary culture techniques

The study protocol used was approved by the Institutional Research Ethics Committee (ERB #1.376.415). The study was conducted according to the tenets of the Declaration of Helsinki. A signed informed consent was obtained from the first-degree families of the corneal donors authorizing their use for treatment or scientific purposes. Moreover, a signed informed consent was obtained from the membrane donor authorizing its use for research purposes.

Ten human corneas discarded for use in corneal transplants due to low endothelial cell counts were used to culture the CEC. The limbal tissues were cultured separately from the corneal buttons, and the Descemet’s membrane, including the endothelial layer, was peeled off. The mean age of the corneal donors was 55.25 years (minimum, 29 years; maximum, 67 years).

Under sterile conditions, in a laminar flow hood, the corneal buttons and limbal tissues were washed with Dulbecco’s Modified Eagle Medium: Nutrient Mixture F-12 (DMEM/F12) (Gibco, Grand Island, NY, USA) with 100 IU/mL penicillin, 40 µg/mL gentamicin, and 2 µg/mL amphotericin-B (Gibco, Grand Island, NY, USA), and were cultured using the following five techniques:

**- Culture of explants in flasks without hydrophilic surface treatment:** The samples were cut into small fragments of approximately 2 mm^3^, which were seeded in 25 cm^2^ culture flasks without hydrophilic surface treatment, for a total of 100 cultures. Each explant was maintained with a drop of bovine fetal serum (BFS) for 30 min, allowing for adherence of the explants to the culture plate, before carefully adding the nutrient medium only to cover the explants, without allowing them to float.
**- Culture of explants in flasks with hydrophilic surface treatment**: The samples were cut into 2 mm^3^ explants, incubated for 30 min with a drop of BFS over each fragment allowing for explant attachment in tissue culture flasks with hydrophilic surface treatment (CellBIND culture flask, Corning, New York, NY, USA). The nutrient medium was added only until the explants were covered, without floating.
**- Culture of explants on amniotic membrane (AM):** The AM was obtained at the time of a cesarean section from a patient with negative serological tests for HIV-1, hepatitis B, hepatitis C, and syphilis. Under sterile conditions, the AM was washed three times using 0.9% physiological solution and once using phosphate buffer solution (PBS) containing 1,000 U/mL of penicillin, 20 mg/mL of streptomycin, and 2.5 mg/mL of amphotericin-B (Gibco, Grand Island, NY, USA). The amnion was separated from the chorion, and the AM was layered over sterile nitrocellulose filter papers of 3 × 3 mm, with the epithelial leaflet facing up. The AM fragments were stored at -80°C in DMEM-F12 nutrient medium with glycerol at a 1:1 dilution. Immediately before use, the AM was thawed for 30 min at room temperature, washed three times using PBS, and incubated for 15 min using 1 ml trypsin-EDTA (Gibco, Grand Island, NY, USA). The amniotic epithelial cells were removed by gentle scraping using cell scrapers. Once de-epithelialization was com-plete, the AM was washed twice using PBS to remove the cell debris and was attached to the bottoms of 35 mm Petri dishes with the basement membrane facing up; this enabled it to act as a substrate for the adherence of explants, which were cut into 2 mm^3^ fragments and placed on the surface.
**- Culture with enzymatic digestion:** The corneal tissues were cut into 2 mm^3^ fragments and were submitted to enzymatic digestion using 0.2% collagenase 2 for 30 min, centrifuged for 10 min at 1,200 rpm, resuspended in 2 ml nutrient medium, and placed in 24-well culture plates.
- **Corneal scraping:** The corneas were scraped with surgical blades to remove the epithelial cells, which were seeded in 25 cm^2^ tissue culture flasks with nutrient medium.

The nutrient medium for the CEC cultures used in all five techniques was Dulbecco’s Modified Eagle Medium: Nutrient Mixture F-12 (DMEM/F12) (Gibco, Grand Island, NY, USA) supplemented with 5 ml/L of TC Minimal Eagle vitamins (Sigma-Aldrich, St. Louis, MO, USA), 0.01 U/mL recombinant human insulin (Gibco, Grand Island, NY, USA), 15 µg/mL glutathione (Sigma--Aldrich, St. Louis, MO, USA), 100 IU/mL penicillin, 40 µg/mL gentamicin, 2 µg/mL amphotericin-B (Gibco, Grand Island, NY, USA), and 20% fetal bovine serum (FBS) (Gibco, Grand Island, NY, USA). The cultures were incubated at 37ºC, with 5% CO_2_, in a humid atmosphere. The nutrient medium was changed every 3 days until reach semi-confluence, occupying 60%-70% of the culture flask, when the CM was collected in each medium exchange until the third passage, filtered with a 0.2 µm micro-filter (Axygen, Glendale, AZ, USA), and stored at 4°C-8°C until use.

### Mesenchymal stem cell primary cultures

The human bone marrow (BM) sample was obtained from a 46-year-old patient, during a surgical procedure for orthopedic humeral osteosynthesis. MSCs were harvested according to Ramakrishnan et al.^(13)^. These cells were characterized to ensure they possessed the phenotypic and functional criteria used to identify MSCs, which include their ability to adhere to plastic surfaces, the expression of specific cell surface antigens, and their potential to differentiate into lineages of mesenchymal tissues, including osteocytes and adipocytes^(4,14)^.

Third-passage MSCs were phenotypically characterized using flow cytometry using the following panel of markers: CD73, CD90, CD105, CD34, CD45, CD11b, CD19, and HLA-DR (BD Stemflow hMSC Analysis Kit, Becton Dickinson Pharmingen, Sparks, MD, USA). Briefly, cells were detached by trypsinization, and 100 µL of the cell suspension at a concentration of 5-10 × 10^6^ cells/mL were dispensed into microtubes and were mixed with monoclonal antibodies or their respective isotypic controls (Table 1) and were incubated for 30 min at 2ºC-8ºC, protected from light. Cells were washed twice using cold PBS containing 10% FBS, were resuspended in 250 µl of the same solution, and were placed in cytometry tubes. Furthermore, data were immediately acquired using a FACS Canto II (BD Biosciences, San Jose, CA, EUA) flow cytometer and were analyzed using the Flow Jo software (Tree Star, Ashland, OR, USA).

**Table 1:** Antibodies used for MSC immunophenotyping

Target	Isotype	Fluorochrome	Clone number	Purpose
CD90	IgG1κ	FITC	5E10	Positive selection
CD105	IgG1κ	PerCP-Cy5.5	266	Positive selection
CD73	IgG1κ	APC	AD2	Positive selection
CD34	IgG2aκ	PE	581	Exclusion marker
CD11b	IgG2aκ	PE	ICRF44	Exclusion marker
CD19	IgG2aκ	PE	HIB19	Exclusion marker
CD45	IgG2aκ	PE	HI30	Exclusion marker
HLA-DR	IgG2aκ	PE	G46-6	Exclusion marker

All the antibodies were supplied by the Stemflow hMSC Analysis Kit (Becton Dickinson Pharmingen, Sparks, MD, USA), including the compensation controls and isotype controls. Irrelevant antibodies were used as isotype controls and for the compensation of the cytometer. These antibodies were provided by the Stemflow hMSC Analysis Kit.

For osteogenic differentiation, 3.1 × 10^3^cells/cm^2^ of third-passage MSCs were plated on a 10 mm round coverslip in four wells of 24-well plate with MSC basal medium (MSCBM) nutrient medium (Lonza, Walkersville, MD, USA) and were cultured at 37°C with 5% CO_2_ for 24 h for cell adhesion before undergoing nutrient exchange. The control wells were filled with MSC basal medium (MSCBM), whereas the cells induced to differentiate received hMSC Osteogenic Differentiation Medium (Lonza, Walkersville, USA) every 3 days for 24 days. Calcium deposition was identified by histochemical staining with Alizarin Red S (Sigma-Aldrich, St. Louis, MO, USA). For adipogenic differentiation, third--passage MSCs at a density of 2.1x 10^4^/cm^2^ were plated in a 24-well plate, on round coverslips as mentioned above. Cells were cultured in MSCBM nutrient medium for 7 days until reaching confluence. Control cultures were kept in MSCGM medium, while adipogenesis was induced by culturing in hMSC Adipogenic Differentiation Medium (Lonza, Walkersville, USA), alternated with MSCGM medium every 3 days for 13 days. Adipogenesis was confirmed using histochemical staining of fat vacuoles with Oil Red O (Sigma-Aldrich, St. Louis, MO, USA).

### In vitro differentiation of MSCs into corneal epithelial cells

MSCs were induced to differentiate into corneal epithelial cells by being cultured with CM derived from primary CEC cultures. For the differentiation assay, 3 × 10^5^ second-passage MSCs were seeded in 25 cm^2^ culture flasks, with nutrient medium for the CEC cultures containing 10% FBS and 40% CM. The cultures were maintained in a humidified incubator with 7% CO_2_ at 37°C, with the medium changed every 3 or 4 days for 21 days. After this period, cells were phenotyped for pan-cytokeratin antibody conjugated to Alexa 488 (Novus Biologicals, Centennial, CO, USA), anti-cytokeratin 3 antibody (CK3) (Abcam, Cambridge, MA, USA) used with the FITC conjugation kit (Abcam, Cambridge, MA, USA), and anti-cytokeratin 12 antibody (CK12) conjugated to PE (LSBio, Seattle, WA,USA) using flow cytometry (Table 2).

**Table 2:** Markers for cytokeratin used to characterize the corneal epithelial cells by immunofluorescence

Specificity	Host	Fluorophore	Dilution	Cat. No and source
pan-cytokeratin	Mouse	Conjugated to Alexa 488	2.5 µl	NBP1-48348AF488; Novus Biologicals, Centennial, CO, USA
CK3	Mouse	FITC conjugation kit	2 µl	ab77869ul100; Abcam, Cambridge, MA, USA ab102884ug300; Abcam, Cambridge, MA, USA
CK12	Rabbit	Conjugated to PE	2 µl	LS-C261556; LSBio, Seattle, WA, USA

Pan-cytokeratin, CK3, and CK12 antibodies used for the characterization of the MSC differentiated into corneal epithelial cells by immunofluorescence.

## RESULTS

### Corneal epithelial cell primary culture techniques

**- Culture of explants in flasks without hydrophilic surface treatment:** After 14 days of proliferation, only five of the 100 cultures had proliferated, producing only 8 ml of CM. The main issue with this technique was the detachment of the explants during the nutrient medium exchange (Figure 1A).
  **Figure 1:**
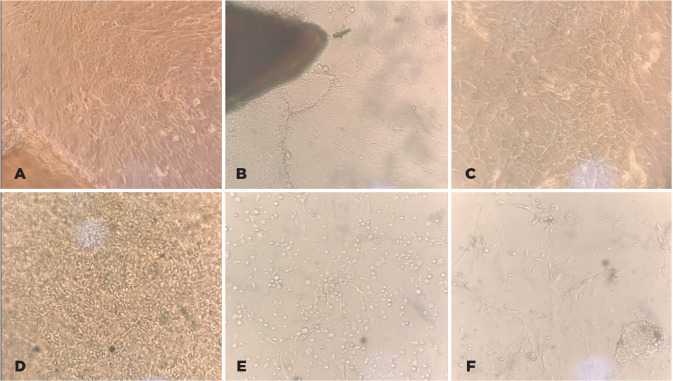
Techniques for corneal epithelial cell cultures. (A) Culture of explants using flasks without hydrophilic surface: cells migrating from explants after 10 days culture (20× magnification). (B) Culture of explants using hydrophilic surface flasks: cells migrating from the explant after 10 days (20× magnification). (C) Detail of the proliferating cells on hydrophilic surface flasks after 14 days (40× magnification). (D) Culture on AM: cells proliferating after 14 days (20× magnification). (E) Culture with enzymatic digestion: cells proliferating after 14 days (20× magnification). (F) Culture by scraping technique: cells proliferating after 14 days (20× magnification).
**- Culture of explants in flasks with hydrophilic surface treatment:** This culturing technique resulted in the highest yield of CM. Each 75 cm^2^ culture flask with proliferating CEC from one cornea produced 20 ml of CM until the third cell passage (Figures 1B and 1C).
**- Explants on AM:** The explants immediately adhered to the AM, with rapid migration and proliferation. However, cellular adhesion to the AM was so intense that the proliferating CEC could not be detached for subsequent cell passages, enabling CM collection only until the first cell confluence (Figure 1D).
**- Culture with enzymatic digestion:** This technique presented spindle-shaped cell types mixed with epithelial cells, probably representing corneal fibroblasts, which impeded the collection of CM due to culture contamination (Figure 1E).
**- Corneal scraping:** This technique resulted in high rates of contamination with corneal fibroblasts and cell debris, which makes collecting CM unfeasible (Figure 1F).

### Mesenchymal stem cell primary cultures

**Figure 2:**
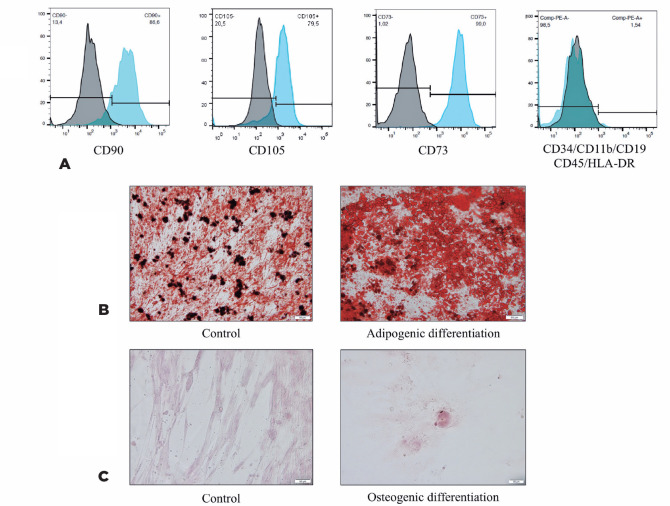
Characterization of MSCs. (A) MSCs were positive for CD90, CD105, and CD73 and were negative for CD34, CD11b, CD19, CD45, and HLA-DR as analyzed using flow cytometry. (B) MSCs induced to adipogenic differentiation showed lipid vesicles in the cytoplasm stained by Oil Red O and the controls stained only the lipid present in the plasmatic membranes of the cells. Scale bar = 50 µm. (C) MSCs induced to osteogenic differentiation stained with Alizarin Red S, showing the formation of bone matrix mineralization nodules, with calcium deposits. Scale bar = 50 µm.

Phenotypic characterization using flow cytometry demonstrated that the analyzed cells were compatible with populations of MSCs, as identified by the characteristics of cell size and granularity. The analyzed cells (Figure 2A) showed high expressions of CD73 (96.1%), CD105 (75.4%), and CD90 (83.5%) and absent or very low expressions of CD34, CD45, CD11b, CD19, and HLA-DR (average, 1.54%). The human MSC population must be highly positive for the surface markers CD73, CD90, and CD105 when measured using flow cytometry. In addition, the markers CD34, CD45, and CD14 must be expressed in less than 2% of the cell population^(14)^; this was used to identify potential contaminants, including hematopoietic stem cells.

MSCs induced to differentiate into adipocytes were also stained using Oil Red O, showing a large number of lipid vesicles in the cytoplasm. The control cells demonstrated only lipids in plasma membranes (Figure 2B). MSCs induced to differentiate into osteocytes were stained using Alizarin Red S, demonstrating the presence of mineralization nodules of the bone matrix, with calcium deposits. The non-induced control maintained the elongated MSC morphology (Figure 2C).

These results allowed us to validate our approach to ensure the obtaining of a homogeneous culture of human bone marrow MSCs.

### MSC differentiation into corneal epithelial cells

**Figure 3:**
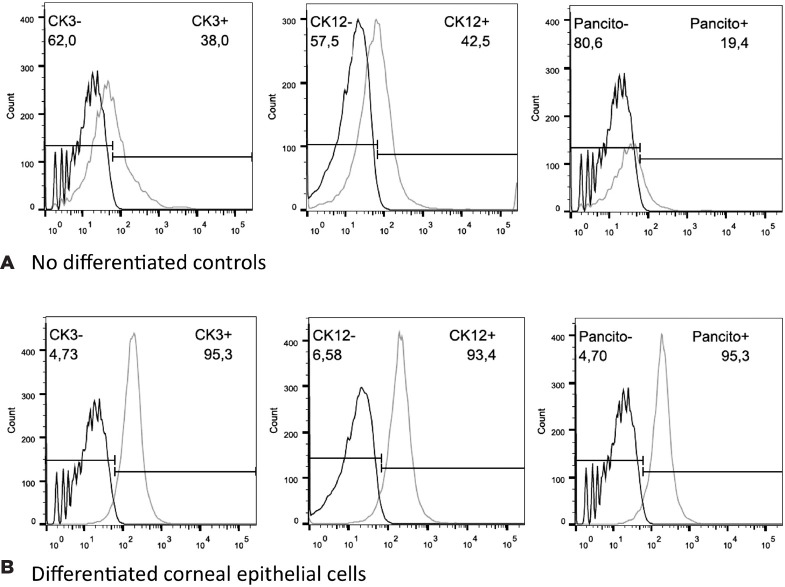
Flow cytometry analysis of MSCs induced to differentiate into corneal epithelial cells labeling for the cornea-specific markers CK3 and CK12. (A) The non-differentiated controls were labeled in 38% and 42.5% for CK3 and CK12, respectively. (B) The differentiation-induced group was labeled in 95.3% and 93.4% for CK3 and CK12, respectively. The pan-cytokeratin marker was labeled 95.3% and 19.4% of the induced and control groups, respectively.

Analysis using flow cytometry showed that MSCs differentiated into CEC, as featured by the expression of corneal cytokeratin-specific markers for CK3 (95.3% of positive cells vs 38% for the control), CK12 (93.4% vs 42.5% for the control), and pan-cytokeratin (95.3% vs 19.4% for the control), confirming the effectiveness of the differentiation protocol (Figure 3).

## DISCUSSION

This study demonstrated that the CEC culture technique using explants in flasks with hydrophilic treatment allowed for rapid and strong explant adherence, with the highest success rate in establishing primary cultures and the highest yield of CM.

Current regenerative medicine has been focused on cell-based strategies, and many previous studies have revealed that cell-based therapy is the most common approach available to replace damaged CEC and regenerate the ocular surface^(15,16)^, with efforts on the development of scaffolds for the cell transference therapy. Bone marrow MSCs are a source of cells with high proliferative capacity that can differentiate into lineages of mesenchymal tissues. However, for the differentiation of MSCs into specialized cells, such as CEC, it requires a reasonable amount of CM. The knowledge of the best technique for the primary culture of CEC to obtain CM is of fundamental importance.

The culture technique of explants in flasks without hydrophilic surface treatment did not allow for the sufficient adherence of tissue fragments to the flasks, which detached during the nutrient medium exchanges. This feature resulted to a very low success rate, making its use unfeasible for the production of CM.

The culture using enzymatic digestion and corneal scraping was shown to be unfeasible due to corneal fibroblast contamination. As the MSCs will be differentiated into CEC by the CM obtained from these cultures, contamination by other cell types is unacceptable.

The technique on AM resulted in rapid and strong explant adherence, confirming that cell adherence and proliferation are easily facilitated by the presence of a basal membrane. However, this technique only allowed CM collecting during the first cell confluence, due to the difficulty in detaching the cells from the AM. If there is no need to detach the cells for further cell passages, as in cases of cell transference using AM as scaffold, this is a very fast and easy execution technique.

The culture of explants in flasks with hydrophilic surface treatment allowed a strong adherence of explants, which did not detach during the nutrient medium exchanges, as seen in cultures in flasks without surface treatment. It also resulted in a high yield of CM, as it was collected until the third cell passage.

The characterization of MSCs before the induction of differentiation showed that these cells strongly expressed CD73 (96.1%), CD105 (75.4%), and CD90 (83.5%) and showed an absence or very low levels of CD34, CD45, CD11b, CD19, and HLA-DR (average, 1.54%). The human MSC population must be highly positive for the surface markers CD73, CD90, and CD105, when measured using flow cytometry. Additionally, the markers CD34, CD45, and CD14 must be expressed in less than 2% of the cell population^(14)^. Staining using Oil Red O and Alizarin Red S showed that these cells were able to differentiate into adipocytes and osteoblasts. This ability to differentiate into adipocytes and osteoblasts combined with the immunophenotyping results allowed us to characterize the cells as human bone marrow MSCs.

After the MSC differentiation and induction into corneal epithelial cells using the CM, the flow cytometry analysis revealed that the induced cells were CEC, as demonstrated by the expression of corneal cytokeratin--specific markers for CK3 (95.3% of positive cells vs 38% for the control), CK12 (93.4% vs 42.5% for the control), and pan-cytokeratin (95.3% vs 19.4% for the control), confirming the effectiveness of the cell differentiation protocol.

This study also demonstrated that the in vitro differentiation of bone marrow MSCs into CEC can be achieved using CEC nutrient medium supplemented with 40% CM. Other authors^(17)^ used CM at a 1:1 proportion.

Our results indicated that culturing of explants in flasks with hydrophilic surface treatment is the most suitable technique to obtain CM collected from primary CEC cultures. Despite that the CM can be useful to induce the differentiation of MSCs into CEC, further studies are needed to confirm the utility of this technique to produce tissues for corneal regeneration.

The best CEC culture technique to obtain CM to induce the differentiation of MSCs into corneal epithelial cells is the culture of explants in culture flasks with hydrophilic surface treatment. This technique produces a high yield of CM, which can be useful to obtain corneal epithelial cultured cells from MSCs.
